# “Loneliness is a monotonous thing”: descriptive qualitative research on the loneliness of caring relatives

**DOI:** 10.1186/s12912-023-01327-4

**Published:** 2023-05-15

**Authors:** Flurina Chistell, Sabrina Stängle, André Fringer

**Affiliations:** 1 Department Internal Medicine, Regional Hospital Surselva, Ilanz, Switzerland; 2 School of Health Professions, ZHAW Zurich University of Applied Sciences, Katharina-Sulzer-Platz, 9, Winterthur, 8400 Switzerland

**Keywords:** Loneliness, Caring relatives, Chronic illness, Qualitative interview, Experiences

## Abstract

**Background:**

The phenomenon of loneliness is increasing worldwide. Caring relatives (CRs) are at high risk of suffering from loneliness. Although some studies have already investigated the issue of loneliness among CRs, there is a lack of evidence to help understand the experience of loneliness in depth. The aim of this study is to record and analyse the experience of loneliness among CRs of chronically ill people. Specifically, the aim is to develop a conceptual model based on the concepts of social, emotional, and existential loneliness.

**Methodology:**

A qualitative-descriptive research design with narrative semistructured interviews was chosen. Thirteen CRs—three daughters, six wives and four husbands—participated in the study. The participants were an average of 62.5 years old. The interviews took place from September 2020 to January 2021 and lasted an average of 54 min. The data were analysed inductively using coding. The analysis was carried out in the following three coding phases: initial open coding, axial coding, and selective coding. The central phenomenon was abductively generated from the main categories.

**Results:**

A chronic illness gradually changes the participants’ normal lives over time. A feeling of social loneliness is experienced, as their quality of social contacts no longer meets their needs. Thoughts about the future and the question of why are omnipresent can create a feeling of existential loneliness. Lack of communication in the partnership or in the family relationship, the changed personality of the ill person as well as the resulting role shift are stressful. Moments of closeness and tenderness become rare, and a change in togetherness takes place. In such moments, there is a strong feeling of emotional loneliness. Personal needs rapidly fade into the background. One’s own life comes to a standstill. Accordingly, loneliness is perceived by the participants to be a stagnant life and is experienced as monotonous and painful. Feelings such as helplessness, powerlessness, frustration, anger, and sadness accompany this loneliness.

**Conclusion:**

The study results show that the feeling of loneliness is present and experienced in a similar way by CRs, regardless of age and relationship to an ill person and that a need for action must derive from this. With the conceptual model, it is possible to offer versatile starting points for nursing practice, such as sensitization, to foster further research into the topic.

## Introduction

The phenomenon of loneliness is increasing in populations worldwide [1, 2]. Loneliness has become important for social and health policy [1]. It is not a purely individual problem, but a social one. It can be described as a taboo subject in which the stigmatization of affected people also plays a role [3, 4]. Loneliness is defined as “(…) an individual, unpleasant and painful feeling arising from unfulfilled or insufficiently fulfilled social and emotional needs related to relationships with other people” [3]. Loneliness can be described as something unpleasant and distressing [5]. The feeling of loneliness is individual, as is a reaction to it [2, 3]. The literature distinguishes three forms of loneliness: social, emotional, and existential loneliness [2, 6–9]. People feel social loneliness when their social contacts do not meet their personal needs [2, 6, 8, 10]. Emotional loneliness is defined as a lack of closeness to a familiar person, a decline in partnership intimacy or a lack of ability to sustain a close relationship [2, 6, 8]. Existential loneliness occurs when people find themselves in a crisis of meaning due to a lack of space for themselves or for their needs and goals [6]. In this study, loneliness is divided into the concepts of social, emotional, and existential loneliness.

Loneliness can affect all age groups [3, 11] and has negative effects on physical and mental health. Different studies show that loneliness has an influence on the development of depressive symptoms, sleep disorders, physical health problems and performance reductions [12–16]. Finally, loneliness is associated with an increased risk of morbidity and mortality [2, 17–21]. A feeling of loneliness can be short-term or persistent [5]. The term social isolation should be distinguished from loneliness. Social isolation is understood as the objective state of being alone or having few social contacts [3, 22]. Social isolation is not synonymous with loneliness [5, 23]. In contrast to objectively measurable social isolation, loneliness is a subjective perception. People can be alone without suffering from loneliness. In contrast, people can feel lonely even when they have a large social network [3]. Consequently, it is not only the presence and frequency of social contacts that matter but also the quality of interpersonal interactions [3].

Changes in familiar life situations can trigger feelings of loneliness [5]. Chronic illnesses, which create critical changes, are a great challenge for those who are affected as well as for their relatives [24]. Experienced losses can occur in physical functionality, relationships, autonomy, life planning, social roles, and identity [24–29]. Often, without consciously realizing it, caring relatives (CRs) increasingly play the role of caregiver and nurturer [30]. Informal help and care from family members plays an important role in the health care of chronically ill persons [31–33]. CRs are indispensable for care in the home [34]. Informal care ranges from administrative and housekeeping work to nursing and caregiving tasks [30]. According to Kaspar et al. [35], there are approximately 580,000 CRs in Switzerland. The burden on a CR is sometimes very high and can have negative health effects [2, 30, 36]. Different studies show that the majority of CRs are physically and psychologically stressed and have a low quality of life [2, 37–39]. Research shows that CRs have a lower subjective well-being than other family members [40, 41]. According to Arrer and Fringer [6], 23% of CRs feel isolated in their caring role. Moreover, the literature indicates that one-third of CRs greatly reduce their social networks by taking on a caregiving role [17, 38]. Their relationship with the person who is dependent on their care and support may also change [2, 24, 27]. Often, the CR’s own occupation is reduced or given up altogether [38]. Given these effects, CRs are at high risk of suffering from loneliness [2, 42]. Although some studies have investigated the issue of loneliness among CRs, there are no results that support a deeper understanding of their experience of loneliness. Based on the findings of this study, health professionals should be able to advise, accompany, and support CRs in a forward-looking, competent, and prudent manner.

## Aim and question

The aim of this study is to record and analyse the experience of loneliness among CRs of chronically ill people. Specifically, the aim is to generate a conceptual model based on the statements of the participants that represents and reconstructs the experiences and the lifeworlds of CRs through its complexity. This conceptual model should serve as an aid to sensitize health professionals to the phenomenon of loneliness so that CRs can be addressed professionally and in line with their needs and suitable interventions can be derived. Health professionals play a central role in recognizing loneliness among CRs. Awareness of this phenomenon in practice should be increased.

Finally, this study aims to lay a foundation for research on the topic of loneliness in Switzerland and to build on it with further research.

The following research question was the basis for this study: How do CRs of homebound chronically ill people experience the phenomenon of loneliness?

## Methodology

### Design

Based on the aim of the study, a descriptive qualitative study design was chosen [43]. This design allows us to explore the phenomenon within the chosen context with limited resources, to identify themes and patterns about the phenomenon of loneliness among CRs of chronically ill people and to achieve the aims of the work [44]. The qualitative descriptive research design makes it possible to capture the experience of loneliness narratively and to describe it in a guideline-based manner [43, 45].

### Recruitment and sampling

Recruitment took place between August and November 2020 via gatekeepers. Outpatient care service organizations in Rhaeto-Romanic Switzerland and German-speaking Switzerland as well as a regional hospital in Rhaeto-Romanic Switzerland were involved to establish access to the members of the study group. The CRs of homebound chronically ill people were the target group of the study, irrespective of chronic illness, type of care, length of care, relationship status or gender. Inclusion criteria for participation in the study were living together in the same household, residence in Rhaeto-Romanic or German-speaking Switzerland and the ability to express oneself in German or Rhaeto-Romanic. CRs who were not able to talk about their experiences were excluded, as were relatives of people without a chronic illness. Participants were invited to participate in the study by nursing professionals from home care services who agreed to serve as gatekeepers for this study. The gatekeepers were guided by the inclusion and exclusion criteria as well as by the assessment of the salient certified nursing professionals regarding the presence of possible loneliness or the existence of an increased risk. Participants were given initial written information and gave their consent to the gatekeepers to be contacted by author FC. Prior to the interviews, an initial telephone contact took place between the first author and the participants.

Heterogeneous sampling was chosen as the procedure [44] to identify different cases. A total of thirteen CRs of homebound chronically ill people were recruited, irrespective of chronic illness, type of care, length of care, relationship status and gender (Table 1). Three daughters, six wives and four husbands participated in the study. One daughter cared for her mother, and the other two daughters cared for their fathers. The participants ranged in age from 31 to 86 years, with the duration of care ranging from 20 months to 25 years. The ill persons suffered from one or more chronic illnesses.

**Table 1:** Sociodemographic data

Properties	Mean value (span)	Frequency (%)
Total		13 (100%)
Participating		
Women Men		9 (70%) 4 (30%)
**Age** (in years)	62.5 years (31–86 years)	
**Caring relative**		
Daughter Wife Husband		3 (24%) 6 (46%) 4 (30%)
**Sick person**		
Father Mother Wife Husband		2 (16%) 1 (8%) 4 (30%) 6 (46%)
**Duration of care**	8.8 years (20 months − 25 years)	

### Ethical considerations

The study was submitted to the Cantonal Ethics Committee Zurich for review and approved (BASEC No. Req-2020-00844). The ethical approach of this qualitative study is based on the Declaration of Helsinki of 2013 [46]. No health-related data were collected. Participation was voluntary, and prior to conducting the interviews, participants were provided with comprehensive information about the aim of researching loneliness among family caregivers via study information letters and additionally verbally. Furthermore, they were informed about the benefits and their right to withdraw from the study at any time without consequences, and how the data would be analysed. All participants had a few days to consider their participation before signing the written informed consent form. The principle of free “informed written consent” was observed [47]. The transcripts were anonymized. The participants were assured of their irreversible anonymity.

### Data collection

Data collection was conducted by the first author through narrative, semistructured individual interviews and was digitally recorded [47]. The semistructured interview guide is based on an integrative literature review that preceded this work and on the research interest of author FC. The interview guide was used solely to guide the researcher during data collection [47]. The interviews took place from September 2020 to January 2021 and lasted between 30 and 90 min (mean 54 min). They were conducted at a location that each participant chose, either at each participant’s home or in a restaurant. Prior to the interviews, the participants were again informed about the study and the data collection procedure; the stage directions such as the process of the interview, time frame, interruptions during emotional challenges, etc. were discussed so that the participants could orient themselves and feel safe in the interview situation [48]. At the beginning of each interview, each participant was motivated to describe his or her experience and the care situation as comprehensively as possible with the following narrative prompt: “First, I would like to learn more about your care situation. Please tell me how you experience everyday care with your relative(s)”. The interview then shifted into an open dialogue using the interview guide. The interview guide contained four thematic clusters: (1) Experiences of the family caregiver; (2) experience of loneliness; (3) strategies to reduce loneliness; (4) recommendations and advice. Each topic cluster had additional items for differentiation. At the end of the interview, each participant had the opportunity to address aspects he or she had not discussed or expectations that he or she had of the interview. Before each interview ended, missing sociodemographic characteristics such as age, gender, relationship to the ill person, occupation, percentage of employment and living situation were recorded. Observations made during the interviews or spontaneous, analytical thoughts were recorded in writing in field notes and memos and were included in the data analysis [49]. Depending on the course of an interview, the first author stayed on site with the participant until his or her emotions had subsided, and he or she were ready to leave from his or her experience. After eleven interviews, a first practical saturation in the analysis process occurred. The saturation was checked and confirmed by means of two additional interviews. These two interviews were included in the analysis. They were not transcribed but were analysed directly via their sound files.

### Data analysis

The data analysis started after the first interview during its transcription. The audio files were transcribed from the Swiss dialect into written German. Care was taken to stay as close as possible to Swiss German to prevent the loss of any relevant statements or values [50]. The interviews conducted in Romansh were translated directly into High German and transcribed. All transcripts were pseudonymized. The transcription rules were based on Dresing and Pehl [51]; in total, seven rules were used.

Data collection and analysis took place in parallel after the first transcript was written. At the beginning of the analysis, the themes were derived from the narrative prompt and from the interview guide and served as preliminary, i.e., ordering, thematic fields. The data were analysed inductively using the coding recommendations of Saldaña [52]. The analysis took place in three coding phases. In the first phase of initial open coding (“first cycle coding”), the transcripts were coded line by line. Initial codes and in vivo codes were formed. In this first step, the first author stayed as close as possible to the original data [52]. The next phase was axial coding (“second cycle coding”). In this process, the open codes were bundled into subcategories by constant comparison, and categories were developed from these [52]. To support the reconstruction of social reality and interpretation, the coding paradigm of Strauss and Corbin [53] was used as an analytical tool to further develop the categories in such a way that their relationships to each other emerged. Following axial coding, the last step in the analytical work was selective coding (“third cycle coding”). Here, the existing categories were further condensed and revised until the main categories could be generated from them and, from these, the central phenomenon that represents the answer to the research question could be obtained [52].

MAXQDA software (Analytics Pro 2020) was used for data management and analysis. MAXQDA also ensured trustworthiness and transparency in the coding [50, 54].

### Quality criteria

The criteria of credibility, comprehensibility, and confirmation according to Lincoln and Guba [49, 55] as well as the criterion of reflective subjectivity according to Kruse [48] were considered. Reflective subjectivity and the criterion of credibility were achieved through critical exchange in the peer group through communicative, collegial validation in each research process and through author FC’s personal engagement with the topic before entering the field [48, 55]. Traceability was achieved through good documentation of the decision-making processes in the research diary, peer group exchanges, and the MAXQDA file that provided insight into the codes [55]. The criterion of confirmation was achieved through the critical assessment of the research process by the last author and by the peer group.

## Results

Based on the analysis, three main categories could be identified: “chronic illness as a challenge to normal life”, “avoiding loss of control as a family member” and “being torn as a caring relative and family member”. The central phenomenon, “stagnant life—expression of loneliness”, was derived from the main categories and represents the answer to the research question.

### Chronic illness as a challenge to normal life

A chronic disease insidiously changes the life that someone is accustomed to. In some situations, the assumption of care and support had already taken place long before the diagnosis. In some cases, the time until a diagnosis was perceived to be very long. This double burden of care and support and not knowing a diagnosis was experienced as very challenging and enervating. The feeling of not being understood, not being taken seriously, and being left alone by one’s social and personal network and salient health professionals characterized this period. According to participants, these aspects are described as moments of loneliness and make it difficult to deal with their situation, leading to uncertainty. Each moment of a definitive diagnosis was experienced differently: from a feeling of relief and certainty to anger, frustration, and fear. Such anger was related to the length of time it took for a diagnosis to be made and to environments and health professionals who, according to the participants, assessed their situations differently. For some participants, a diagnosis was life-changing, as they now had certainty about the severity of the disease.

The trajectory of a chronic disease is characterized by alternating phases of stability and instability and has been described as complex and difficult to assess. The course of a disease is also characterized by incidents with physical effects:

“That’s 15 years, (…). He has had pneumonia, certainly four, five pneumonias and all sorts of other things. He’s been everywhere, in hospitals, in rehabilitation clinics. (…) But now, of course, it’s much worse than then.“ (I4-071020: 46, wife).

The participants reported that they sometimes felt overwhelmed and powerless during phases of instability. In such moments, some participants lacked any exchange with familiar people or people who could offer them support in their decision-making process. According to the participants, these moments of loneliness were strongly dependent on the course of a disease.

The participants completed different nursing and caring tasks. Depending on the course of an illness, additional tasks could be added, often over time and without the participants consciously realizing it. Suddenly, they seem to be doing everything, performing medical and nursing tasks, housekeeping chores, administrative and coordinating activities, and making control calls while being the contact person continuously. As a result of these increasing activities and their double burden, some participants had reduced their workloads or even stopped working. In addition to these effects on their daily work, the participants reported that they had greatly reduced their meetings with friends, club attendances, sporting activities and travel. In some cases, this led to social isolation and a feeling of social loneliness, as the quality of social contacts no longer met the participants’ needs. External service providers, such as home care services, were involved to varying degrees in care situations. These home care services were experienced differently. Some participants felt that the support was sufficient, valuable, and supportive. Individuals discussed how they grew to become a family with the specialist team involved. Others felt insufficiently supported by the external service providers, left alone and not understood. This was perceived to be stressful. Furthermore, some participants emphasized that they felt lonely despite a social environment and the support of health professionals because the sick person was always the centre of attention:

“(…) I have the feeling that I am only informing, all the time. Either I have to say how she is, or I have to say what to do (…).“ (I8-061120:57, daughter).

Based on the analysis, it became apparent that some CRs feel supported, and others do not. There is no underlying professional attitude toward loneliness on the part of home care providers here, but rather it is a matter of building relationships over time, which has a positive effect on the experience and can be interpreted as familiarity. The participants experience their care situation as very changeable, as their need for support varies. They experience this as a difficult navigation between promoting resources and taking over all the tasks. The majority experience the time required for care as very intensive. The participants describe their care situations as “always being there”. Each care situation is also largely experienced as physically demanding, complex and very stressful. A sick person is partly perceived to be a burden. Thoughts, even outside the home, constantly revolve around the sick person at home. The participants often report feelings of stress, frustration, disappointment, fear, and anger. Feelings of guilt and self-reproach, for example, for not having reacted earlier, are frequent companions. Thoughts about the future and the question why are omnipresent and create a feeling of existential loneliness:

“It just goes, it just has to go. So I don’t get depressed or lonely, I just don’t think about all that could come or all that is coming. I think like the Italians are, today is today and tomorrow you see then. I have adopted that a little bit from them. That’s the best way, otherwise you drive yourself crazy.” (I7-041120, wife).

Their feeling of having reached their limit is palpable. Participants report that they have reached the end of their patience or that they have cried out to God for help. Some also expressed a desire to end this stressful situation by entering a retirement and nursing home:

“At that moment, I thought I had to separate, to send him to a nursing home. Not like that. It was quite bad.“ (I5-091020:73, wife).

A high sense of responsibility and duty also leads to strain and overload. Often, the participants bear all responsibility. Their inhibition threshold for seeking and accepting help is high. Unsatisfactory experiences with external service providers also have a negative influence on their decisions. Often, maintaining freedom and flexibility in daily life is more important than the relief provided by external service providers. Fear of losing privacy is another reason for refusing external help, as is a lack of consent from their ill person.

In the interviews, it becomes clear that in addition to negative aspects, positive issues are also experienced. Taking over care can trigger personal pride, have a positive learning effect, and strengthen one’s personality because of what has been achieved.

### Avoiding loss of control as a family member

The participants have developed strategies for dealing with their caregiving situation, their personal needs, and their own experiences of stress. They want to defend themselves against any threat that could fragment their lives with their ill person and their own routines. This entails denying the threat of loneliness:

In the end, he (the partner in need of care) could no longer allow intimacy. “It hurt somewhere. But I also knew it was good for both of us to let go. That was a big loneliness gap for me. I’ve had two choices, either I give in to this loneliness and I get depressed or fall into an addiction, whatever, or I stand up and say no, I take my life in my own hands (…).“ (I10-191120, wife).

According to some participants, requesting and accepting help is of central importance to their care situation. This means calling external service providers, such as home care services, making use of relief services such as respite care and, if necessary, requesting professional help for themselves. Participants see the added value of support for not feeling alone in their care and being able to share responsibility. This has an impact on the experience of emotional loneliness, since a professional caregiver participates in home care, and over time a personal familiar relationship emerges, which is experienced as relieving. They receive a moment of free personal time from the services involved. This free time reduces their feeling of loneliness for a short moment.

“Also with the home care service, with these women who come to us, we have a friendly relationship that I also (…) every time I see them, I am happy to see them, this exchange with each other. These also share something with us. This is also so valuable. I am very grateful; they are an important cornerstone in our lives.” (Interview 11_201120, wife).

Participants also report that they receive help in their decision-making process and support for maintaining their personal roles as CRs. Furthermore, external service providers provide a sense of security and can help counteract prevailing loneliness.

To a certain extent, according to the participants, their dyad can also compensate for their experience of loneliness. Consciously enjoying time together, exchanging experiences and rediscovering closeness with each other are strategies that help reduce the feeling of emotional loneliness. Other strategies for combatting loneliness are to maintain social contacts, even if time is scarce, and to return to one’s usual leisure activities, if possible. If this is not possible, there is nothing left but to abandon one’s usual routine and look for alternatives that lead to social contacts. In this context, the participants emphasized that it is above all about exchanges with each other:

“If I don’t keep up the social contacts such as singing, music rehearsals, and jassas, one day I’ll be all alone. (…) What is possible, you must keep up (…) even if you no longer have so much time. (…) That is the only thing (…) not to get lonely. (…) sometimes you have to steal the time.“ (I7-041120: 36, wife).

An existing professional activity makes it possible for participants to forget their home for a short time and to maintain social contacts and should therefore be maintained. Some participants recommend the installation of an alarm clock, which allows a certain amount of freedom. Pets can compensate for a lack of closeness to a certain extent and are considered helpful to combat the feeling of loneliness:

“Then I’m back home, then it’s on here, making fire, doing household chores, bedding, laundry, you know, just the way it is. I still look at the cats and at the ducks, of course that’s something that doesn’t give me work, I love that. Otherwise, I wouldn’t have the ducks.” (I4_071020: 14, wife).

In regard to strategies for dealing with one’s own demands, a distinction is made between strategies that relieve stress and those that cause illness. Putting aside one’s own perfectionism, setting priorities and good daily planning help meet one’s own demands. One participant has moved her original meetings to her own’s home so that she can maintain their necessary weekly exchanges and thus counteract social loneliness. For some participants, setting priorities led them to ignore their own needs altogether. The consequence of this was a lack of self-care and less time and space for oneself, which increased the feeling of loneliness:

“In terms of loneliness, right now it’s like I can’t tell if I’m okay or not right now. I’m functioning, I’m not doing too badly, but I couldn’t say I’m doing very well either. My feeling is that my life is passing me by. The gynaecologist told me three weeks ago that my biological clock is ticking. I have my age breathing down my neck.“ (Interview 8-061120, daughter).

According to the participants, faith and personal rituals can serve as sources of strength when dealing with their personal experiences of stress. Discussing one’s own feelings and experiences of stress, whether with one’s immediate environment or with relevant health professionals, are strategies that help counteract the feeling of loneliness. Some participants also mentioned distraction through occupation as another strategy.

### Being torn as a caring relative and family member

The disjointedness in each participant’s experience is expressed when he or she finds himself or herself in the role of carer instead of his or her original role as spouse or daughter. In doing so, they increasingly play the role of carer(s), although they vehemently try to maintain their original role. The participants feel morally obliged to take over care as well as to care. In these conflicts, they have different experiences of dependency. Their ill person seems to be dependent on them, while being a relative depends on the course of the chronic illness and on the consent of the ill person. The situation is very stressful, and in some cases, there is discussion of a strong overload. Some participants report withholding information from their family to protect them and not to burden them. This in turn promotes their feeling of being alone in their situation. In such moments, the participants lack persons who they can contact for advice and exchange. This in turn increases their feelings of loneliness:

“But he says “I’m not going to the nursing home.“ Now I don’t know how to do that. By force, I don’t want to put him in the home. I talk to him again. I had already talked to him once, there he had refused. Now I am alone. (…) I think that I will stick to my decision. I’ll do it. It’s that loneliness you have when it comes to that. I can’t always listen to my kids, they don’t have him around. The youngest son says then I’m here alone. Then I said ‘I don’t mind at all’.“ (I4-071020, wife).

The effect of a chronic illness leads to a conflict between the person with the illness, who tries to maintain own autonomy, and the threat of dependency, which they must increasingly surrender to. This conflict is most apparent when an ill person struggles for his or her autonomy, resists external help, and constantly tries to control his or her treatment. Externally, the ill person signals normality in order to avoid additional impulses to surrender his or her autonomy. According to the participants, this led to being misunderstood or derided by their environments, which in turn reinforced their feelings of loneliness.

The participants experience the physical, cognitive, and psychological changes that occur in the person with a disease. A lack of communication in their relationship, a changed personality of their ill person and participants’ own changed role modify their normal life together. Female participants often speak of having a maternal role, which means that the role of wife or daughter is only partially possible. A chronic illness thus creates dependency:

“The very difficult balancing act between being a lover and being a carer and his dependence on me.“ (I5-091020: 60, wife).

Moments of closeness and tenderness become rare, and a change in togetherness takes place. The participants experience a change in the attentiveness of their partner or parent. Meanwhile, the participants lack collective emotional participation with their ill person. The participants report feeling lonely at such moments. Due to the progression of a disease, their usual joint activities are no longer possible or require a great deal of organizational effort. According to the participants, a chronic illness led to a change in their family. Some have reported that they have had to make major changes to their normal life with their ill person and to their own life because of a chronic illness. They were confronted by existential questions:

“She (the neighbour) is very helpful. Otherwise, I have others as well. I have to tell them because I feel alone, because I don’t have love. I want so much to have someone hug me, kiss me, physical closeness, not sexual, not at all. There is just a lack of physical closeness. That is very, very important for me. When I feel so alone, it almost physically hurts me. Jessas God that can’t be, why me, so many years, so many years, that you don’t have that.“ (I2-190920, wife).

### Central result: Stagnant life—expression of loneliness

**Fig. 1:**
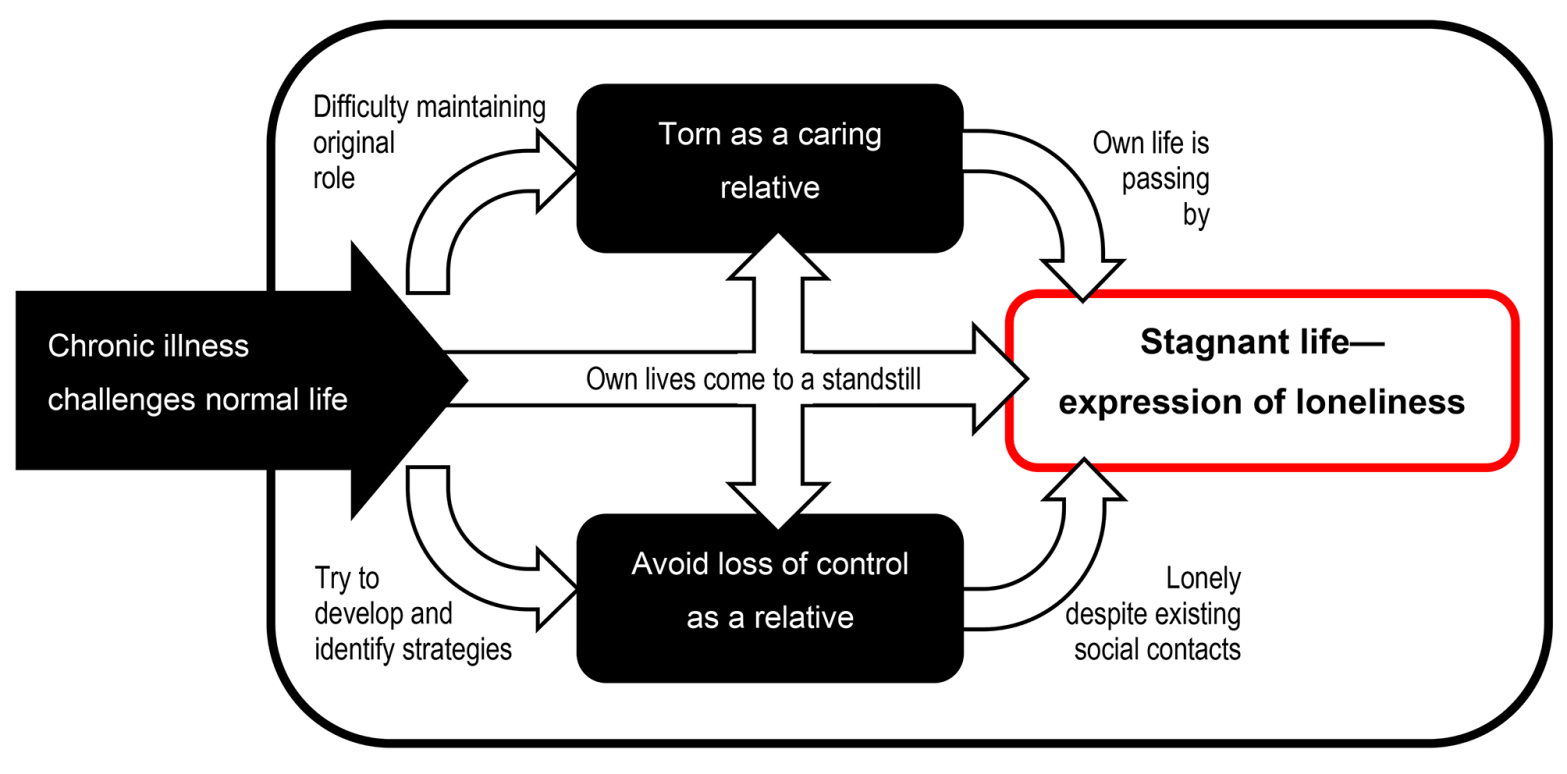
Conceptual model of loneliness as a caring relative: Stagnant life—expression of loneliness

The following chapter describes the central phenomenon, derived from the main categories through the process of selective coding. The conceptual model that has been created represents the experiences of the interview participants (Fig. 1).

A chronic disease and its effects sunder participants from their normal lives. Chronic illness is described as a challenge to one’s normal life. Moments of powerlessness, exchange lacks, and shifting roles as partner or parent create feelings of loneliness. Slowly, CRs become caring and nurturing relatives. In the process, they experience a sense of being torn. Instead of their original role as spouse, partner, daughter, or son, they now find themselves in the role of carer and caregiver. It is considered difficult to maintain their original role. A sense of duty, love and affection for the sick person or a lack of alternatives are the reasons for taking over care and nursing. Existential questions, lacks of closeness and tenderness and lacks of exchange, recognition and appreciation create a feeling of loneliness. The participants are in a constant process of adaptation. They have to deal with chronicity and develop strategies to deal with it. They try to maintain their usual daily routine. In addition to their new tasks of care and support, the participants try to develop strategies to continue to meet their own needs. Despite their attempts to maintain control, many participants report that their needs and their normal life have been set aside due to the chronic illness of their partner or parent. Some discuss having given up everything in their own lives because of a chronic disease:

“I have the feeling that I have given up everything. (…) There is nothing left.“ (I8-061120: 26, daughter).

Their situation does not allow them to live their own lives. Some of the participants feel that their own life is passing them by. They experience changes in their normal everyday life and their usual leisure activities. They lack the time to fulfil their needs. There is also a great need to exchange experiences with people who are in a similar situation. Although self-help groups are a possibility, they often do not work because there are only a few and existing offers do not seem to fit into the individual’s everyday life from the participants’ point of view. A lack of time is the only reason for not satisfying one’s own needs; there is also a lack of energy. The participants also lack free space for themselves. They feel restricted by their constant presence. Often, they no longer dare to be away from home for long. Taking over care can also have an impact on their job. A reduction in workload or dismissal can result. The participants report how they had to surrender their normal everyday life, their needs, their wishes, their dreams and sometimes, even their partner or parent. Their own lives come to a standstill. Loneliness is defined by the participants as a life that has come to a standstill and is painful:

“When I feel so alone, it almost physically hurts me.“ (I2-190920: 61, wife).

It became obvious in the statements of the interview partners that this was not a temporary loneliness, but a chronicized experience. Some participants emphasize that they have been suffering from loneliness for years; their loneliness grew gradually. Some participants also report that loneliness is strongly dependent on the course of a disease. The participants find themselves alone and lonely in their decision-making processes, in administrative matters and when processing what they have experienced. Some participants report feeling lonely despite existing social contacts. One participant described loneliness as follows:

“Loneliness is a monotonous thing.“ (I3-290920: 7, husband)

Feelings such as helplessness, powerlessness, frustration, anger, fear and sadness can accompany this state of suspended animation. Being able to talk to someone, have time for oneself and experience closeness and appreciation are the needs of these participants in a state of suspended animation:

“Sometimes, I think, Jesus, that would be nice if I could talk this through with someone.“ (I3-290920:30, wife)

Notably, the participants want social exchanges with their immediate environment, health professionals and like-minded people. A social environment, as support, is longed for.

## Discussion

The aim of the present study was to investigate the experience of loneliness among CRs of chronically ill people.

Due to a chronic disease’s effects and changes, CRs are sundered from their normal lives [24]. These CRs are in a constant process of adaptation. The analysis shows that the majority of participants consider themselves alone and lonely. Loneliness is described by the participants as monotony and is experienced as something that hurts physically. The literature describes that the same brain areas are activated during loneliness as during physical pain [56, 57]. Thus, loneliness can be understood as a kind of pain. The results show that loneliness is often accompanied by feelings such as helplessness, powerlessness, fear, frustration, anger and sadness. The reason for these feelings was often described as the changeable course of their relative’s disease and its effects on their shared life and on the CR’s life. The phenomenon of loneliness appears not only when a partner or parent is no longer there but also during the time of care. The analysis shows that one’s feeling of loneliness depends on the course of a disease. Loneliness is experienced in a similar way regardless of one’s age and relationship to a sick person.

The analysis highlights the three forms of loneliness mentioned at the beginning of this study: social, emotional, and existential loneliness. Participants often lost or consciously reduced their social contacts. External commitments, such as pursuing one’s own occupation, were also reduced or completely abandoned. Their social network becomes fragile not only because they take complete care of their partner or parent but also because they lack energy [7]. One participant gave an impressive account of how she invited her colleagues to her home so that she could continue to maintain her social contacts and meet her need for exchange. In a study by Vasileiou et al. [2], this was described as an important intervention when one’s radius of action seems to shrink. Vasileiou et al. [2] thus suggested that CRs feel increasingly constricted and restricted due to their loved one’s increasing need for support. The results show that the participants leave their house less and less due to their concerns and guilty consciences. Accordingly, their social contacts continuously dwindle, and the participants increasingly suffer from their feeling of social loneliness. They lack exchanges with people, whether to talk about their situation, ask for advice, or as a diversion and distraction. The need to exchange with people who are in a similar situation was immense. Any offer of a self-help group was often absent but was considered desirable by the majority of participants. Even when many different service providers are involved, most CRs ultimately feel abandoned. They feel that they are not involved enough in decisions and that any focus is mainly on their sick person. They also felt that they were not understood, taken seriously, or noticed. Their desire for a supportive professional relationship at eye level, which enables exchange, counselling, and accompaniment, was thus somewhat longed for. The importance of this need is confirmed by the study of Lindahl et al. [58] and the report of Haslbeck et al. [24], which show that a shared responsibility can minimize the prevailing pressure on CRs.

Participants also reported a feeling of loneliness despite their social contacts. The literature describes how CRs can feel lonely despite their social contacts, as the quality of each social relationships is crucial [3, 11, 21, 23].

Due to an increasing need for care and the resulting dependency, a CR’s relationship with his or her partner or parent changes, which leads to emotional loneliness. It is painful when, after many years of living together, togetherness increasingly dwindles and a change in the ill person’s personality is witnessed [25]. Another factor that leads to emotional loneliness is a change in one’s own role. Participants often discuss playing a maternal role, which means that their role as wife or daughter is only partially possible. These role changes can create changes in their relationship [25, 59, 60]. CRs experience a change in attentiveness and a lack of appreciation of their partner, spouse, or parent. In this context, CRs have often described a changed personality of their ill person. CRs miss the emotional participation of their ill person in their common life. A need for esteem, closeness and tenderness was frequently mentioned, and its satisfaction was rare or completely lacking. These results correspond with the studies of Brügger et al. [31], Plöthner et al. [61], and Vasileiou et al. [2].

However, the analysis also shows that some CRs experience an intensification of their relationship. In these situations, their experience of loneliness is compensated to a certain degree by their dyad. Furthermore, the present study also shows that a supportive relationship with the treatment team could counteract emotional loneliness of CRs [62, 63].

The results show that some participants live only in their present; they report having no future prospects. In this existential loneliness, their own needs and goals are not sufficiently perceived. Several CRs discuss impressive stories of having given up everything because of the chronic illness of their partner or parent. The high demands on their time and their lack of any opportunity to pursue their own interests have a stressful effect and can lead to frustration.

### Relevance for practice

This study aims to increase awareness among health professionals about the phenomenon of loneliness among CRs of chronically ill people. Health professionals play a central role in recognizing loneliness in CRs. Knowledge about CRs’ experience of loneliness is essential to be able to support and accompany them at an early stage and in a way that suits their needs. Based on the findings of this study, health professionals can specifically address CRs and identify possible stressors to aid them in their new roles and counter the phenomenon of loneliness. CRs should be offered existential conversations to address their worries and needs. The development of a supportive relationship is essential.

Since loneliness can develop in all phases of the course of any disease, it is necessary to sensitize a sick person and his or her CR(s) to the topic as early as the initial diagnosis. The existing expertise, experience and developed strategies of CRs should be recognized and valued. The aim is to strengthen CRs through counselling and to support them in developing individual coping strategies for everyday life. When doing so, it is important to work in an interdisciplinary way, whether with specialized professionals or within self-help groups. Moreover, many CRs have a high inhibition threshold in regard to asking for help. For this reason, it is extremely important to increase the awareness of CRs to help them request and accept help early enough and without feelings of guilt, and this must be addressed and supported by health professionals.

### Relevance for research

Both social and emotional loneliness have been explored to some degree. However, considering the present study, it is necessary to take a closer look at the issues of disenfranchised loss and loneliness due to changing roles in the partnership from the perspective of the CRs. In this context, loneliness due to personality changes of the ill family member should be examined more closely from the CRs perspective in comparison to the experiences of other family members and professional outpatient caregivers. Especially the aspect of “intra-family injustice” in role attribution and role adoption as a CRs should be more studied.

This study has laid a foundation for further research on loneliness in Switzerland. The findings from this study apply to both Rhaeto-Romanic and German-speaking Switzerland. Based on these findings, it would be important and opportune to develop a questionnaire for a standardized survey to obtain an overall picture of the phenomenon of loneliness among CRs of chronically ill people in Switzerland.

### Limitations

A strength of this study is its descriptive, qualitative research design. It enabled an exploration of the phenomenon and an inductive approach with limited resources. The goal of heterogeneous sampling was achieved and made it possible to deeply analyse a broad spectrum of lived experiences regarding the phenomenon of loneliness and to synthesize them into a conceptual model. The cultural aspects of Rhaeto-Romanic and German-speaking Switzerland should be considered if the conceptual model is to be transferred to other settings. Another limitation of this study is that no statement can be made about possible variations in the experiences of loneliness with respect to the phases of a chronic illness, and due to the small sample, no differences in these experiences regarding age, relationship status or length of care could be analysed. The recruitment process is another limitation. Concerning concept development, it would have been important to achieve theoretical sampling and to focus on a grounded theory study design. To achieve this, recruitment would have needed to be expanded. Following the final two interviews, there were signs of practical saturation, but due to the sample size, theoretical saturation could not be assumed.

With this study, a conceptual model could be developed that allows further research on the topic. Support by peers and regular exchanges with the supervisors of this research can be emphasized as further strengths. The chosen quality criteria according to Lincoln and Guba as well as Kruse were considered in this work.

## Conclusion

Important findings on the phenomenon of loneliness were identified. These study results show that loneliness is present, regardless of a CR’s age and relationship to an ill person, that it is experienced similarly by CRs, and that a need for action must be derived from this. Loneliness is defined by a CR as a stagnant life. A feeling of social loneliness is experienced because a CR’s quality of social contacts no longer meets his or her needs. Thoughts about the future and the question of why are omnipresent and create a feeling of existential loneliness. Lack of communication in a CR’s relationship with the changing personality of an ill person amid a CR’s own shifting role modify their shared life. Moments of closeness and tenderness become rare, and a change in togetherness takes place. In such moments, there is a strong feeling of emotional loneliness.

This study serves to sensitize health professionals to the phenomenon of loneliness among CRs of chronically ill people. With the conceptual model, it is possible to offer versatile starting points for nursing practice and for further research on the topic.

## Data Availability

The datasets consist of thirteen qualitative participant interviews. These are confidential and according to the guidelines of the ethical approval not accessible to the reader. Requests to access the data should be made by contacting the corresponding author.

## Abbreviations

CR: Caring Relatives
